# Hyponatremia in Patients with Spontaneous Intracerebral Hemorrhage

**DOI:** 10.3390/jcm3041322

**Published:** 2014-11-20

**Authors:** Jaime Robenolt Gray, Kathryn A. Morbitzer, Xi Liu-DeRyke, Dennis Parker, Lisa Hall Zimmerman, Denise H. Rhoney

**Affiliations:** 1 Department of Pharmacy, Hospital of the University of Pennsylvania, Philadelphia, PA 19104, USA; E-Mail: jaimeleigh03@gmail.com; 2 Division of Practice Advancement and Clinical Education, UNC Eshelman School of Pharmacy, University of North Carolina at Chapel Hill, Chapel Hill, NC 27599, USA; E-Mail: kmorbitzer@unc.edu; 3 Department of Pharmacy, Detroit Receiving Hospital, Detroit, MI 48201, USA; E-Mails: xiliu@yahoo.com (X.L.-D.); dejue@wayne.edu (D.P.); 4 Department of Pharmacy Practice, Eugene Applebaum College of Pharmacy and Health Sciences, Wayne State University, Detroit, MI 48201, USA; 5 Department of Pharmacy, New Hanover Regional Medical Center, Wilmington, NC 28401, USA; E-Mail: lisahallzimmerman@gmail.com

**Keywords:** hyponatremia, intracerebral hemorrhage, syndrome of inappropriate antidiuretic hormone, cerebral salt wasting syndrome

## Abstract

Hyponatremia is the most frequently encountered electrolyte abnormality in critically ill patients. Hyponatremia on admission has been identified as an independent predictor of in-hospital mortality in patients with spontaneous intracerebral hemorrhage (sICH). However, the incidence and etiology of hyponatremia (HN) during hospitalization in a neurointensive care unit following spontaneous intracerebral hemorrhage (sICH) remains unknown. This was a retrospective analysis of consecutive patients admitted to Detroit Receiving Hospital for sICH between January 2006 and July 2009. All serum Na levels were recorded for patients during the ICU stay. HN was defined as Na <135 mmol/L. A total of 99 patients were analyzed with HN developing in 24% of sICH patients. Patients with HN had an average sodium nadir of 130 ± 3 mmol/L and an average time from admission to sodium <135 mmol/L of 3.9 ± 5.7 days. The most common cause of hyponatremia was syndrome of inappropriate antidiuretic hormone (90% of HN patients). Patients with HN were more likely to have fever (50%* vs.* 23%; *p* = 0.01), infection (58%* vs.* 28%; *p* = 0.007) as well as a longer hospital length of stay (14 (8–25)* vs.* 6 (3–9) days; *p* < 0.001). Of the patients who developed HN, fifteen (62.5%) patients developed HN in the first week following sICH. This shows HN has a fairly high incidence following sICH. The presence of HN is associated with longer hospital length of stays and higher rates of patient complications, which may result in worse patient outcomes. Further study is necessary to characterize the clinical relevance and treatment of HN in this population.

## 1. Introduction

Hyponatremia is the most frequent electrolyte abnormality in hospitalized patients, especially those with neurologic injury, and is associated with increased morbidity and mortality [1,2]. Hyponatremia in patients with neurological injury may exacerbate cerebral edema through fluid shifts causing intracranial hypertension and potentially leading to worsening of neurological outcomes. The in-hospital mortality in brain-injured patients with severe hyponatremia (serum sodium <130 mmol/L) has been reported to be 50 times higher than patients with normal sodium levels [3]. Studies have also demonstrated prolonged lengths of stay in neurosurgical patients with hyponatremia by as many as 7 days [4,5,6,7]. Early identification and correction of hyponatremia is essential in these patients in order to prevent additional complications or worsening of outcomes [8].

While hyponatremia has been widely described after traumatic brain injury, subarachnoid hemorrhage, and neurosurgical intervention, there is limited information describing the incidence and etiology of hyponatremia in patients with spontaneous intracerebral hemorrhage (sICH). Previous studies assessing the pathogenesis of hyponatremia following subarachnoid hemorrhage [4,5,6,7] or traumatic brain injury [9] have implicated the syndrome of inappropriate antidiuretic hormone (SIADH) or cerebral salt wasting syndrome (CSWS). In addition to determining the incidence, it is also crucial to determine the etiology of hyponatremia in patients following sICH because these two syndromes are treated differently; SIADH is generally treated with fluid restriction, while CSWS is treated with sodium containing fluids. Thus, misdiagnosis can lead to worsening of hyponatremia and potentially worsening of neurological outcome [10].

Given the clinical importance of hyponatremia in other neurological disease states, we were interested in evaluating hyponatremia following sICH as, to our knowledge, it has not previously been elucidated. We conducted a retrospective analysis at our institution with the primary objective to identify the incidence of hyponatremia following sICH. Secondary objectives included determining the etiology of hyponatremia in patients with sICH as well as the consequences of hyponatremia in these patients.

## 2. Experimental Section

### 2.1. Patients

This was a retrospective analysis conducted at Detroit Receiving Hospital. All patients admitted to the neurocritical care unit at Detroit Receiving Hospital between January 2006 and July 2009 with sICH, identified by ICD-9 diagnosis code 431, were eligible for inclusion. The diagnosis of sICH was validated in these patients through review of the both the electronic and paper medical records and confirmation through radiological evidence.

### 2.2. Patient Demographics and Clinical Variables

Medical records were retrospectively analyzed for the following baseline patient characteristics: age, gender, race, past medical history, social history, baseline metabolic panel and complete blood count, medications used prior to admission, Glasgow Coma Scale (GCS) at admission, location of sICH, sICH volume, and surgical interventions for the management of sICH. Data collected relating to the diagnosis and management of hyponatremia included: serum sodium and osmolality during the entire hospitalization, urine electrolytes and osmolality, brain naturietic peptide (BNP), thyroid stimulating hormone (TSH), cortisol, duration and severity of hyponatremia, timing from sICH onset to development of hyponatremia, mannitol and hypertonic saline use, concomitant medications known to effect sodium regulation, fluids/colloids given, central venous pressure (CVP), fluid status, and blood pressure. Clinical outcome measures collected include: hospital and intensive care unit (ICU) length of stay, in-hospital mortality, and complications associated with hospital course.

### 2.3. Study Definitions

Hyponatremia was defined as serum sodium <135 mmol/L [11]. The distinction between CSWS and SIADH is often very difficult to assess in the clinical setting, and may therefore be unreliable. The differentiation between CSWS and SIADH in this study was determined using previously identified criteria from the literature. SIADH was defined as euvolemic hyponatremia with inappropriate urine concentration (urine osmolality > serum osmolality), and low urine volume with natriuresis [12]. In contrast, CSWS was defined as hypovolemic hyponatremia as identified by low CVP, with diuresis (urine output >250 mL/h) and natriuresis [13]. In the absence of a CVP monitoring device, fluid balance and/or volume status were assessed within 48 hours of the development of hyponatremia where negative fluid balance was defined as greater than 1000 mL negative; neutral to positive fluid balance was defined as negative 1000 mL to positive 1000 mL; and positive fluid balance was defined as greater than 1000 mL positive. Patients who received diuretics within 48 hours of hyponatremia were not evaluated for pathogenesis.

### 2.4. Statistical Analysis

Descriptive statistics were used to describe the patients included in this study. Normonatremic and hyponatremic patients were compared using Student’s* t*-test or Mann-Whitney *U* test for continuous variables and Fisher’s exact test for categorical variables. Continuous data were described using mean ± SD or median (interquartile range) depending on the distribution of the data. Results were considered significant when the *p*-value <0.05. SPSS v. 17.0 for Mac (SPSS Inc., Chicago, IL, USA) was utilized for all statistical analysis.

## 3. Results and Discussion

### 3.1. Incidence

A total of 258 patients were coded with a diagnosis of ICH. The same diagnosis code was given for patients who experienced a sICH, trauma induced intracerebral hemorrhage, anticoagulation induced intracerebral hemorrhage, or subdural hematoma. Only patients with a complete record who experienced a sICH were included in this study. Following case review for verification of this criteria, 99 patients were included in this study. Twenty-four (24%) patients developed hyponatremia following sICH. Patient characteristics are presented in Table 1. Overall there were no differences in baseline demographic variables or disease severity characteristics between the patients who developed hyponatremia and those who did not.

**Table 1:** Baseline Patient Characteristics: CKD, chronic kidney disease; ESRD, end stage renal disease; NSAIDs, nonsteroidal anti-inflammatory drugs; COX, cyclooxygenase; SSRI, selective serotonin reuptake inhibitor; GCS, Glasgow Coma Scale; ICH, intracerebral hemorrhage; HN, hyponatremia.

Characteristic	No HN (*n* = 75)	HN (*n* = 24)	*p*-Value
Age, years, mean ± SD	58.6 ± 10.4	59.4 ± 12.1	0.73
Male gender, *n* (%)	43 (57)	18 (75)	0.12
African American, *n* (%)	59 (77)	17 (75)	0.71
Past Medical History, *n* (%)			
Neurological Injury *	14 (19)	1 (4)	0.11
Seizure Disorder **	1 (1)	1 (4)	0.43
Hypertension	59 (79)	20 (83)	0.77
Diabetes	17 (23)	4 (17)	0.53
Heart Failure	8 (11)	1 (4)	0.45
CKD or ESRD	16 (21)	2 (8)	0.23
Social History, *n* (%) **			
Tobacco	24 (32)	10 (42)	0.39
Alcohol	19 (25)	10 (42)	0.13
Illicit Drugs	22 (29)	5 (21)	0.81
Medications Prior to Admission			
Diuretic	13 (17)	3 (13)	1.0
NSAIDs and COX-2 Inhibitors	3 (4)	1 (4)	1.0
SSRI	1 (1)	0 (0)	1.0
Laboratory Parameters on Admission			
Sodium (mmol/L)	143 ± 3	140 ± 3	0.002
Potassium (mEq/L)	4.1 ± 0.8	4.2 ± 0.7	0.63
Serum Creatinine (mg/dL)	1.7 ± 1.83	1.11 ± 0.52	0.13
Glucose (mg/dL)	145 ± 66	155 ± 70	0.51
GCS, median (IQR)	14 (10, 15)	14 (7, 15)	0.85
Location of ICH, *n* (%)			
Lobar	36 (48)	11 (46)	0.85
Deep	28 (37)	13 (54)	0.15
Brainstem/Cerebellar	11 (15)	0 (0)	0.06
Surgical Intervention, *n* (%)			
Craniectomy	1 (1)	3 (13)	0.01
Craniotomy	2 (3)	1 (4)	0.67
Stereotaxy Aspiration	1 (1)	0 (0)	0.58
ICH Volume (mL), median (IQR)	22 (8, 44)	32 (9, 89)	0.30

* Defined as any type of past stroke or traumatic brain injury, as recorded in the medical record; ** Variable collected if any history of seizures or seizure disorder, tobacco use, alcohol use, or illicit drug use as recorded in the medical record.

### 3.2. Description of Hyponatremia

**Figure 1:**
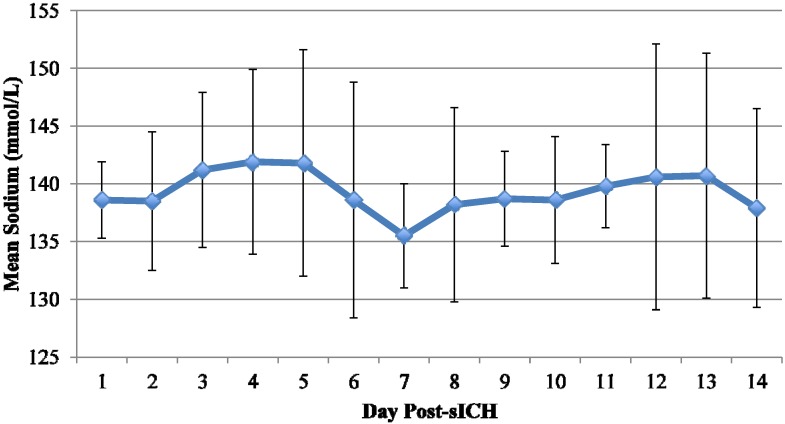
Mean sodium following sICH in patients with hyponatremia.

**Figure 2:**
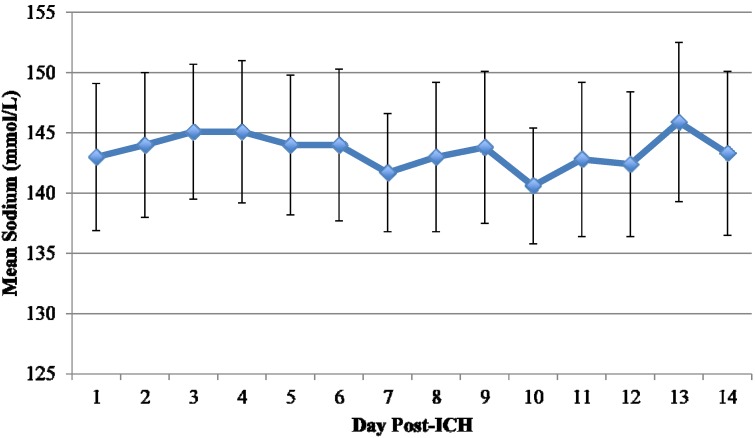
Mean sodium following sICH in patients without hyponatremia.

Variables describing hyponatremia are represented in Table 2. Patients who developed hyponatremia had lower sodium on admission than those who did not develop hyponatremia (140 ± 3.3* vs.* 143 ± 3.3 mmol/L; *p* = 0.002). Nine patients who had hyponatremia developed severe hyponatremia, defined as serum sodium <130 mmol/L. The mean time from admission to sodium concentration <135 mmol/L was 3.9 ± 5.7 days. As expected, patients who developed hyponatremia had a lower mean sodium nadir (130 ± 3* vs.* 139 ± 3 mmol/L; *p* < 0.001) and lower mean sodium throughout hospitalization (139 ± 4* vs.* 143 ± 4 mmol/L; *p* < 0.001). Figure 1 and Figure 2 display the mean sodium concentrations following sICH in patients who developed hyponatremia and those who did not. Of the 24 patients who developed hyponatremia, 15 (62.5%) developed it within the first week following sICH. The remaining 9 patients developed hyponatremia ≥8 days following sICH. The median time for normalization of sodium in patients with hyponatremia was 48.6 hours; however, 5 of the 24 patients (20.8%) did not resolve their hyponatremia.

**Table 2:** Description of hyponatremia (HN).

Variable	No HN (*n* = 75)	HN (*n* = 24)
Sodium Nadir (mmol/L), mean ± SD	139 ± 3	130 ± 3
Average Sodium (mmol/L), mean ± SD	143 ± 4	139 ± 4
Time from Admission to Sodium <135 mmol/L (days), mean ± SD	-	3.9 ± 5.7

### 3.3. Etiology of Hyponatremia

Of the 24 patients who developed hyponatremia, 20 were evaluated for etiology. The etiology of HN was SIADH in 18 patients with 2 of these cases thought to be drug related due to carbamazepine. The other 2 patients were determined to have developed hyponatremia secondary to CSWS.

### 3.4. Clinical Outcomes, Complications, and Mortality

Clinical outcomes and complications are represented in Table 3. Patients who developed hyponatremia had an increased rate of infection (58%* vs.* 28%; *p* = 0.007), fever (50%* vs.* 23%; *p* = 0.01), and thrombocytopenia (17%* vs.* 1%; *p* = 0.01). Patients who developed hyponatremia also had an increased length of hospital stay (14 (8, 25)* vs.* 6 (3, 9) days; *p* < 0.001); however, there was no difference in length of ICU stay between the two groups (10 (7, 20)* vs.* 4 (2, 9) days; *p* = 0.91). In-hospital mortality among patients who developed hyponatremia was 25%, which was not significantly different than patients who did not develop HN (32%; *p* = 0.52).

**Table 3:** Clinical outcomes, ICU complications, and ICU patient medications: HN, hyponatremia; LOS, length of stay; ICU, intensive care unit; ACEi, angiotensin converting enzyme inhibitor.

Variable	No HN (*n* = 75)	HN (*n* = 24)	*p*-Value
Clinical Outcomes			
Hospital LOS (days), median (IQR)	6 (3, 9)	14 (8, 25)	<0.001
ICU LOS (days), median (IQR)	4 (2, 9)	10 (7, 20)	0.91
In-Hospital Mortality, *n* (%)	24 (32)	6 (25)	0.52
Complications, *n* (%)			
Seizures	4 (5)	3 (13)	0.36
Cerebral Edema	43 (57)	17 (71)	0.24
Fever	17 (23)	12 (50)	0.01
Infection	21 (28)	14 (58)	0.007
Thrombocytopenia	1 (1)	4 (17)	0.01
Inpatient Medications, *n* (%)			
Furosemide	19 (25)	14 (58)	0.01
ACEi	30 (40)	15 (63)	0.21
Carbamazepine	0 (0)	2 (8)	0.06
Antidepressants	2 (3)	0 (0)	1.0
Opioids	2 (3)	1 (4)	0.57
Hypertonic Saline	3 (4)	2 (8)	0.62
Mannitol	8 (11)	2 (8)	0.72

Table 3 also lists inpatient medication regimens. Patients who developed hyponatremia were more likely to have been administered furosemide (58%* vs.* 25%; *p* = 0.01) and two patients who developed hyponatremia were also administered carbamazepine. The administration of these medications may have resulted in a medication-induced hyponatremia. All patients received 0.9% sodium chloride as IV fluids, if necessary, and as the standard diluent for IV medications when compatible.

### 3.5. Discussion

This study is the first to demonstrate the incidence, etiology, and clinical characteristics of patients who develop hyponatremia after admission following sICH. A prominent percentage of patients in our study developed hyponatremia (24%), as defined by Na <135 mmol/L, with SIADH appearing to be the predominate cause. As the development of hyponatremia may lead to worse neurological outcomes and as those who developed hyponatremia in our study had an increased length of hospital stay, these represent important clinical findings for this patient population.

Previous studies in acute subarachnoid hemorrhage (aSAH) and traumatic brain injury (TBI) have demonstrated that hyponatremia (Na <135 mmol/L) occurs anywhere from 13% to 58% and severe hyponatremia (Na <130 mmol/L) occurs in 15% to 19.6% of patients [4,6,7,14]. In terms of the timing of the development of hyponatremia, the majority of patients developed hyponatremia in the first 3 days following aSAH, although it was observed that patients were still developing hyponatremia >7 days after injury [4,7,9].

The incidence of hyponatremia in our study was 24%. While this is comparable to the incidence observed in patients with TBI, it is lower than what has been reported in patients with aSAH. We also had a very low rate of severe hyponatremia, defined as Na <130 mmol/L, (8 patients (8.1%)) as compared to the aSAH studies. The differences in our low rates could be attributed to the standard of practice in our neurocritical care unit of ensuring that all IV fluids administered are sodium chloride and that all IV medications have sodium chloride as the diluent where compatibility allows. Although we did not evaluate the treatment of hyponatremia in these patients (due to the limitations of the retrospective nature of the study), hypertonic saline infusions are commonly utilized at our institution for the treatment and/or prevention of increased intracranial pressure due to cerebral edema. Therefore, pre-emptive administration of these infusions may have contributed to the low incidence observed in our study. In addition, fluid restriction was not specified for any patients in this study.

Of clinical importance, hyponatremia occurred not only within the first week following sICH, but 9 patients (37.5%) developed hyponatremia ≥8 days following sICH. This finding is similar to that observed in the aSAH and TBI populations, as a significant number of those patients also developed hyponatremia >7 days from injury. The timing of the development is important to consider when monitoring patients, both as an inpatient or for outpatient follow-up, to prevent further neurological insult secondary to hyponatremia.

In our study, we found that SIADH was the most common cause of hyponatremia. This is an important distinction because CSWS has been implicated in many patients with hyponatremia following aSAH [7]. Distinguishing the etiology is essential since treatment may vary, where SIADH can be treated with fluid restriction or vasopressin-receptor antagonists while aggressive intravenous sodium administration is the treatment of choice for patients with CSWS.

To our knowledge, only 1 study has evaluated hyponatremia in the sICH population. Kuramatsu and colleagues investigated the prevalence and clinical associations of hyponatremia on admission in patients with sICH [15]. They found that the prevalence of hyponatremia on admission was 15.6% and in-hospital mortality was doubled in patients who presented with hyponatremia compared to those who did not (40.9%* vs.* 21.1%). This further highlights the importance of identifying patients with hyponatremia. Our study expands on these results by highlighting the incidence and timing of the development of hyponatremia in patients with sICH through their hospital course.

In previous studies, patients who developed hyponatremia had a longer length of stay and worse clinical outcomes [4,9,15]. This is similar to our findings as patients in our study had a significantly longer hospital length of stay and subsequently had higher rates of complications, such as fever and infection. Although patients with hyponatremia in our study did not have an increased rate of cerebral edema or seizures, this may be due to the small number of patients in our study who developed severe hyponatremia (Na <130 mmol/L), which has been shown to cause these complications [4]. The increased length of stay and ICU complications observed may be associated with hyponatremia, or its treatment, or may be a result of the severity of the underlying illness that is then associated with the development of hyponatremia and other complications. This is of clinical importance because the impact of longer hospital/ICU admission can be multi-factorial and result in worse patient outcomes, as well as increased costs and resource allocation [16,17]. These may serve as future outcome measures to determine the impact of longer lengths of stay in patients who develop hyponatremia.

Our study has several limitations, primarily its single-center inpatient retrospective design, which limited the patients available for analysis. This then resulted in only a small insight into the etiology of hyponatremia and the complications associated with it in this patient population, as the longer-term implications of treatment and resolution could not be elucidated. Another limitation of our study is that SIADH and CSWS are often difficult to differentiate in a clinical setting. Not all patients included in this study had CVP measurements and event with CVP measurements, determining the etiology of SIADH if not absolute. In the absence of CVP measurements, fluid balance was used in the distinction of SIADH from CSWS. This practice is also frequently required in the clinical setting as well. Therefore, our study does shed more light on the issue of hyponatremia in a real-world practice setting, and provides further insight into the complications associated with patients who develop hyponatremia in this population.

## 4. Conclusions

Hyponatremia following sICH is a common electrolyte abnormality that has been previously underreported and may result in worsening of patient outcomes. Patients should be monitored closely following sICH for trends in sodium levels. This study adds to the limited data describing hyponatremia in patients with sICH. Future studies are necessary to evaluate the role and impact of additional sodium variables may play on hyponatremia, as well as clinical complications and outcomes for these patients. Results from future studies could provide information on how to safely and effectively provide the best patient monitoring for hyponatremia in this patient population as well as optimal therapeutic interventions.
